# “Non-criteria” antiphospholipid antibodies add value to antiphospholipid syndrome diagnoses in a large Chinese cohort

**DOI:** 10.1186/s13075-020-2131-4

**Published:** 2020-02-21

**Authors:** Tingting Liu, Jieyu Gu, Liyan Wan, Qiongyi Hu, Jialin Teng, Honglei Liu, Xiaobing Cheng, Junna Ye, Yutong Su, Yue Sun, Jinfeng Zhou, Gary L. Norman, Xuefeng Wang, Chengde Yang, Hui Shi

**Affiliations:** 1 Department of Rheumatology and Immunology, Ruijin Hospital, Shanghai Jiao Tong University School of Medicine, No. 197 Ruijin Second Road, Huangpu District, Shanghai, 200025 China; 2 Werfen China, 10 Jiuxianqiao RD., Chaoyang District, Beijing, China; 3 INOVA Diagnostics, Inc., 9900 Old Grove Road, San Diego, CA 92131 USA; 4 Department of Laboratory Medicine, Ruijin Hospital, Shanghai Jiao Tong University School of Medicine, Shanghai, China

**Keywords:** Antiphospholipid syndrome, Non-criteria antiphospholipid antibodies, Anti-phosphatidylserine/prothrombin antibodies, Anti-β2-glycoprotein I Domain 1, IgA isotype

## Abstract

**Background:**

Despite expansion in the 2006 Sydney antiphospholipid syndrome (APS) classification criteria to include IgG/IgM anti-β2-glycoprotein (aβ2GPI) antibodies in addition to IgG/IgM anti-cardiolipin antibodies (aCL) and lupus anticoagulant (LAC), some individuals with clinical features of APS remain seronegative (seronegative APS or SNAPS) and are at risk of recurrent thrombosis and pregnancy morbidities. Our aim was to assess the value of “non-criteria” aPL antibodies to detect these SNAPS patients.

**Methods:**

One hundred ninety-two APS patients, 90 SNAPS patients, 193 autoimmune disease controls, and 120 healthy controls were evaluated. Ten antiphospholipid antibodies (aPLs) were tested using commercial kits, including 5 non-criteria aPLs: anti-phosphatidylserine/prothrombin antibodies (aPS/PT) IgG/IgM, aCL IgA, aβ2GPI IgA, and anti-β2GPI Domain 1 (aβ2GPI-D1) IgG.

**Results:**

Up to 60.9% of the SNAPS and 93.5% of APS patients were detected by at least one non-criteria aPL. aPS/PT IgG had the highest Youden index in classifying APS and SNAPS from controls. aPS/PT IgG and aβ2GPI Domain 1 IgG seem to be the most significant risk factors for thrombotic events and pregnancy morbidity, respectively. aPS/PT IgG/IgM and aβ2GPI-D1 IgG were detected in some SNAPS patients, while IgA isotypes of aCL/aβ2GPI tended to appear together with other biomarkers. The combined analysis showed enhanced diagnostic performance with the inclusion of non-criteria aPLs.

**Conclusions:**

Recognition of SNAPS patients is critical for clinical management and prevention of potential thrombotic and obstetric adverse events. The non-criteria antiphospholipid antibodies help to identify a considerable portion (60.9%) of these patients who otherwise may remain untreated and at clinical risk.

## Background

The antiphospholipid syndrome (APS) is an autoimmune disease characterized by recurrent arterial and/or venous thrombosis, pregnancy morbidity, and persistent presence of antiphospholipid antibodies (aPLs). APLs have served as important serological markers in the diagnoses of APS since the syndrome was first described by Hughes in 1983 [1]. The original APS “Classification Criteria” included only anti-cardiolipin (aCL) IgG/IgM and lupus anticoagulant (LA) as accepted laboratory criteria [2]. In 2006, the sensitivity of the classification criteria was improved by the inclusion of aβ2GPI IgG/IgM antibody and the specificity was improved by extending the requirement of persistent presence to 12 weeks [3]. Still, in clinical practice, patients exist with typical clinical manifestations highly suggestive of antiphospholipid syndrome but remain persistently negative for criteria aPLs. These patients have been termed seronegative APS (SNAPS) by Hughs and Khamashta [4].

SNAPS patients, just as classic APS patients, manifest increased risk for thrombotic events and pregnancy morbidities. These events may reoccur during the natural course of the disease [5] and in rare cases can result in a life-threatening thrombotic state leading to multi-organ dysfunction known as catastrophic antiphospholipid syndrome (CAPS) [6–8]. Identification of patients with SNAPS, maintaining regular follow-up and providing therapeutic or preventative medication, remains a significant challenge.

Increased recognition and a deeper understanding of APS have evolved with the development of assays for non-criteria aPLs and research into their role in the pathophysiology of APS. These non-criteria aPLs, which include anionic phospholipids, phospholipid-protein complexes, and plasma proteins, may help physicians to better manage suspected APS patients [9]. For example, research on anti-prothrombin (aPT) antibodies eventually led to the recognition of the importance of the complex of phosphatidylserine and prothrombin as a target for aPL antibodies.

Anti-phosphatidylserine/prothrombin antibodies (aPS/PT) are now acknowledged as a highly effective potential marker for APS [10, 11]. As a result of its remarkable diagnostic performance and high prevalence in the LA-positive patient group, as validated by multiple studies [12–14], aPS/PT has gained much attention and was included in both the APS-S (Otomo) and the Global APS Score (GAPSS), widely used systems for patient thrombotic risk assessment [15]. Additional biomarkers continue to be evaluated for their value to APS diagnosis and management. The development of assays to specifically examine domain-specific β2-glycoprotein antibodies have highlighted the pathogenic role of anti-β2GPI Domain 1 (D1) IgG antibodies [16–18], in contrast to anti-domain 4/5 IgG antibodies which have been characterized as “innocent” antibodies [19]. The IgA isotypes of aPLs are not included as criteria biomarkers; however, they are recommended as adjunctive biomarkers for individuals suspected of APS, but negative for conventional biomarkers [20, 21]. A recent study confirmed the extra predictive value of IgA isotypes when both IgG and IgM were negative [22]. Still, other aPLs, including anti-annexin 5 antibody, anti-protein C [23], anti-protein S [24], anti-vimentin/cardiolipin complex [25], and anti-lysobisphosphatidic acid (LBPA) [26], also been suggested as relevant new biomarkers, but their value in APS diagnosis and management is still unclear. In the last few years, new methodological approaches, including thin-layer chromatography (TLC) [27, 28], multiline dot assay [29], and chemiluminescence [30], have been developed for refining laboratory diagnosis of APS. The antigenic presentation of proteins and/or phospholipid/protein complexes are quite different in these three systems as compared to standard ELISA, and they might be useful to expand our knowledge on the antigen specificities of “antiphospholipid antibodies.”

In the current study, five of the most promising non-criteria antibodies for which commercial kits were widely available, namely aCL IgA, aβ2GPI IgA, aPS/PT IgG/IgM, anti-β2GPI Domain 1 (aβ2GPI D1) IgG, were selected for evaluation. The value of these markers to assist in the detection of SNAPS patients and their value in predicting in adverse clinical events as assessed by odds ratio (OR) were analyzed in a large cohort of APS and seronegative patients, as well as in patients with a variety of other autoimmune diseases and healthy adults as controls. The individual and the combined value of the non-criteria antibodies in classifying APS, as well as SNAPS patients, were then evaluated.

## Methods

### Patients recruitment

A total of 595 patients were included in this case-control study. The research was performed according to the Declaration of Helsinki and approved by the Institutional Review Broad of Ruijin Hospital (ID: 2016-62), Shanghai Jiaotong University School of Medicine, Shanghai, China. Informed consent was obtained from all individuals included in this study. Patients were classified into seven subgroups:

-Group 1: 192 APS patients from the APS-Shanghai (APS-SH) database, which was established by expert rheumatologists and statisticians at the Shanghai Jiao Tong University School of Medicine (Shanghai, China). All patients satisfied the 2006 Sydney classification criteria. In this group, 88 patients were classified as primary antiphospholipid syndrome (PAPS) and 104 patients as secondary antiphospholipid syndrome (SAPS) patients, of which 76 were comorbid with SLE, 25 with lupus-like disease, 2 with Sjogren’s syndrome, and 1 with rheumatoid arthritis.

-Group 2: 90 SNAPS patients from the APS-SH database fulfilling the Sydney clinical criteria but persistently negative for criteria aPLs. Diagnoses were established and confirmed by at least two expert rheumatologists in accordance with the definition by Hughs and Khamashta [4], and hereditary and other acquired thrombophilia were excluded before patients were enrolled. In addition to the major clinical criteria that fulfilled the Sydney classification criteria for APS, at least one of the non-criteria manifestations associated with APS were also required to make the diagnosis of SNAPS. The non-criteria clinical features are as follows: (1) superficial vein thrombosis, (2) thrombocytopenia, (3) renal microangiopathy, (4) heart valvular disease, (5) livedo reticularis, (6) migraine, (7) chorea, (8) seizure, (9) longitudinal myelitis, (10) epilepsy, (11) Raynaud’s phenomenon, and (12) brain MRI showed white matter lesions [5, 31]. More details about this group of patients were added in the text as thrombocytopenia was recorded in 33.33% (30/90) SNAPS. A history of heart valvular disease and livedo reticularis were frequently documented in SNAPS (21.11% (19/90) and 17.78% (16/90), respectively). There are 16.67% (15/90) patients with brain MRI showed white matter lesions. 8.89% (8/90) of patients in the SNAPS group developed Raynaud’s phenomenon. Migraine was found in 7.78% (7/90) SNAPS patients. And epilepsy is recorded in 4.44% (4/90) patients. There are 9 patients with two of these manifestations.

-Group 3: 103 patients with systemic lupus erythematosus (SLE) satisfying both the revised 1997 American College of Rheumatology(ACR) set of classification criteria [32] and the 2012 criteria established by the Systemic Lupus International Collaborating Clinics (SLICC) group [33].

-Group 4: 29 patients with Sjogren’s syndrome (SS) satisfying the ACR/EULAR 2016 classification criteria [34].

-Group 5: 30 patients with ankylosing spondylitis (AS) satisfying the 1984 revised New York Criteria [35].

-Group 6: 31 patients with rheumatoid arthritis (RA) satisfying ACR/EULAR 2010 classification criteria [36].

-Group 7: 120 healthy controls (HC), without autoimmune, neoplastic or infectious diseases.

Demographic characteristics of the cohort are depicted in Table 1. All patients were Chinese and were enrolled continuously from 2000 to 2017. Complete medical histories, laboratory tests, and medical images were recorded in the database and all patients underwent regular follow-up either by the outpatient department or by telephone follow-up. Thrombotic events and pregnancy morbidities (PM) were recorded. Thrombosis was classified into arterial thrombosis and venous thrombosis, while pregnancy morbidities were divided into early and late pregnancy. Early pregnancy morbidity referred to at least one fetal loss before 10 weeks of pregnancy while late pregnancy morbidity referred to at least one stillbirth after 10 weeks of pregnancy or premature delivery before 34 weeks of pregnancy. The occurrence of stroke and deep vein thrombosis was also recorded. Serum samples were collected and frozen at − 80 °C until testing. Diagnoses were confirmed by at least two rheumatologists from Shanghai Jiao Tong University School of Medicine. Serum levels of the criteria aPLs, except for lupus anticoagulants, were determined by semi-quantitative QUANTA Lite® ELISA kits provided by Inova Diagnostics, Inc. (San Diego, CA, USA). Lupus anticoagulant was detected and evaluated by the clinical laboratory of Ruijin Hospital according to the ISTH recommendations. The assay was performed at the patient’s first visit to the rheumatology department and repeated 12 weeks later.

**Table 1:** Demographic characteristics and antibody profile of APS and SNAPS patients

	APS	SNAPS	SLE	SS	RA	AS	HC
Numbers	192	90	103	29	31	30	120
Age (Q1–Q3)	35 (30–46)	39 (32–51)	34 (29–43)	42 (35–51)	47 (40–58)	34.5 (27–40)	39 (34–46)
Sex (F/M)	162/30	77/13	92/8	27/2	28/3	15/15	108/12
Arterial thrombosis	74 (39%)	53 (59%)	0 (0%)	0 (0%)	0 (0%)	0 (0%)	0 (0%)
Venous thrombosis	78 (41%)	33 (37%)	0 (0%)	0 (0%)	0 (0%)	0 (0%)	0 (0%)
Early fetal loss	36 (22%)	12 (16%)	0 (0%)	0 (0%)	0 (0%)	0 (0%)	0 (0%)
Late fetal loss	81 (50%)	18 (23%)	0 (0%)	0 (0%)	0 (0%)	0 (0%)	0 (0%)
aCL IgG/IgM(+)	115 (60%)	0 (0%)	6 (6%)	0 (0%)	0 (0%)	0 (0%)	0 (0%)
aB2GPI IgG/IgM(+)	130 (68%)	0 (0%)	13 (13%)	0 (0%)	0 (0%)	1 (3%)	0 (0%)
LA(+)	133 (69%)	0 (0%)	6 (6%)	0 (0%)	0 (0%)	0 (0%)	0 (0%)
aCL IgA(+)	80 (42%)	11 (12%)	12 (12%)	0 (0%)	0 (0%)	0 (0%)	0 (0%)
aB2GPI IgA(+)	75 (39%)	9 (10%)	7 (7%)	0 (0%)	0 (0%)	0 (0%)	0 (0%)
aPS/PT IgG(+)	137 (71%)	32 (36%)	15 (15%)	1 (3%)	0 (0%)	0 (0%)	0 (0%)
aPS/PT IgM(+)	141 (73%)	32 (36%)	33 (32%)	7 (24%)	0 (0%)	3 (10%)	5 (4%)
aB2GPI D1 IgG(+)	119 (62%)	14 (16%)	7 (7%)	0 (0%)	0 (0%)	0 (0%)	2 (2%)

### Laboratory tests

#### Anti-cardiolipin antibodies (aCL) and anti-β2-glycoprotein I antibodies (aβ2GPI)

aCL IgG/IgM/IgA and aβ2GPI IgG/IgM/IgA were measured using QUANTA Flash® (Inova Diagnostics Inc., San Diego, CA, USA) chemiluminescent immunoassays run on the BIO-FLASH® instrument (Biokit s.a., Barcelona, Spain). The cutoff values for the antibodies were defined as 20 chemiluminescent units (CU) as recommended by the manufacturer.

#### Anti-phosphatidylserine-prothrombin antibodies (aPS/PT)

Anti-PS/PT IgG and IgM antibodies were measured using semi-quantitative QUANTA Lite® ELISA kits (Inova Diagnostics Inc., San Diego, CA, USA). The cutoff values for both aPS/PT IgG and IgM were defined as 30 units (U) as recommended by the manufacturer.

#### Anti-β2-glycoprotein I Domain 1 antibodies (aβ2GPI D1)

aβ2GPI D1 IgG was measured using QUANTA Flash® (Inova Diagnostics Inc., San Diego, CA, USA) chemiluminescent immunoassay. The cutoff value was ≥ 20 CU as defined by the manufacturer.

### Statistical analysis

Statistical analysis was performed using R (version 3.5.1). In descriptive statistics, data were expressed in the form of positive numbers, percentages for categorical variables, and median (Q1–Q3) for continuous variables. Chi-Squared test and Fisher’s exact were used to compare the categorical variables, and the Wilcoxon test and Kruskal-Wallis test were used to compare the continuous variables after normality was explored with the Shapiro-Wilk test. Logistic regression models with adjusted gender and age information were used to calculate the odds ratios between different serum markers and clinical events. The outcome of the ORs is presented together with the 95% Wilson confidence interval (CI). The level of statistical significance was set at a two-tailed α-value of 0.05 by default. Diagnostic performances of the different biomarkers were evaluated by calculating the sensitivity, specificity, positive predictive value (PPV), negative predictive value (NPV), positive likelihood ratio (PLR), and negative likelihood ratio (NLR). Both the APS and SNAPS groups are considered the experimental group when evaluating the diagnostic indexes using fourfold table. Relationships between aPL profiles and clinical events are presented as odds ratios.

## Results

### Distribution of antiphospholipid antibodies

**Fig. 1:**
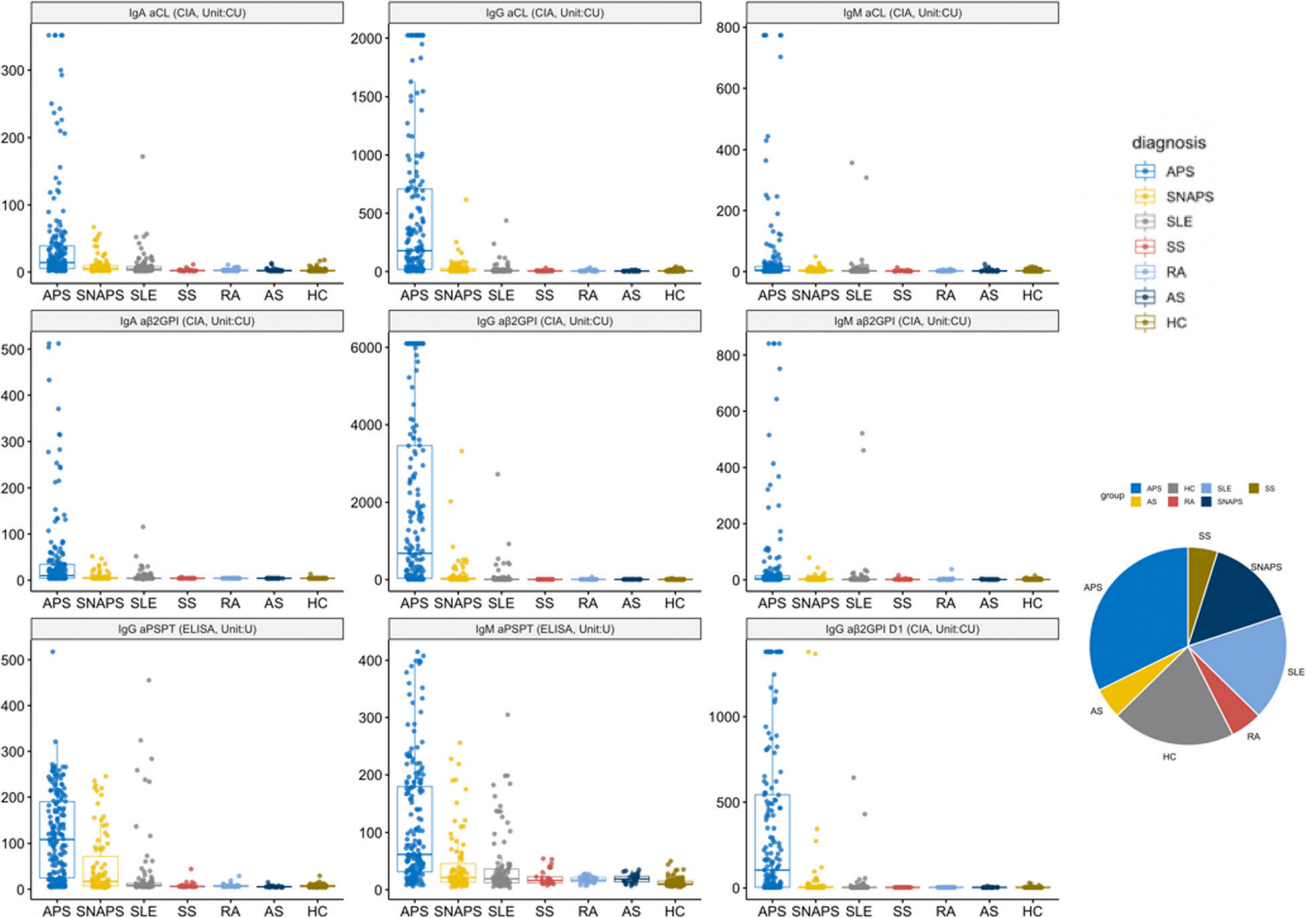
Antibody profile of aPL in APS, SNAPS, and control patients. Dot plot of the nine aPL titers among different diagnostic groups, with the box showing the quantile values

**Fig. 2:**
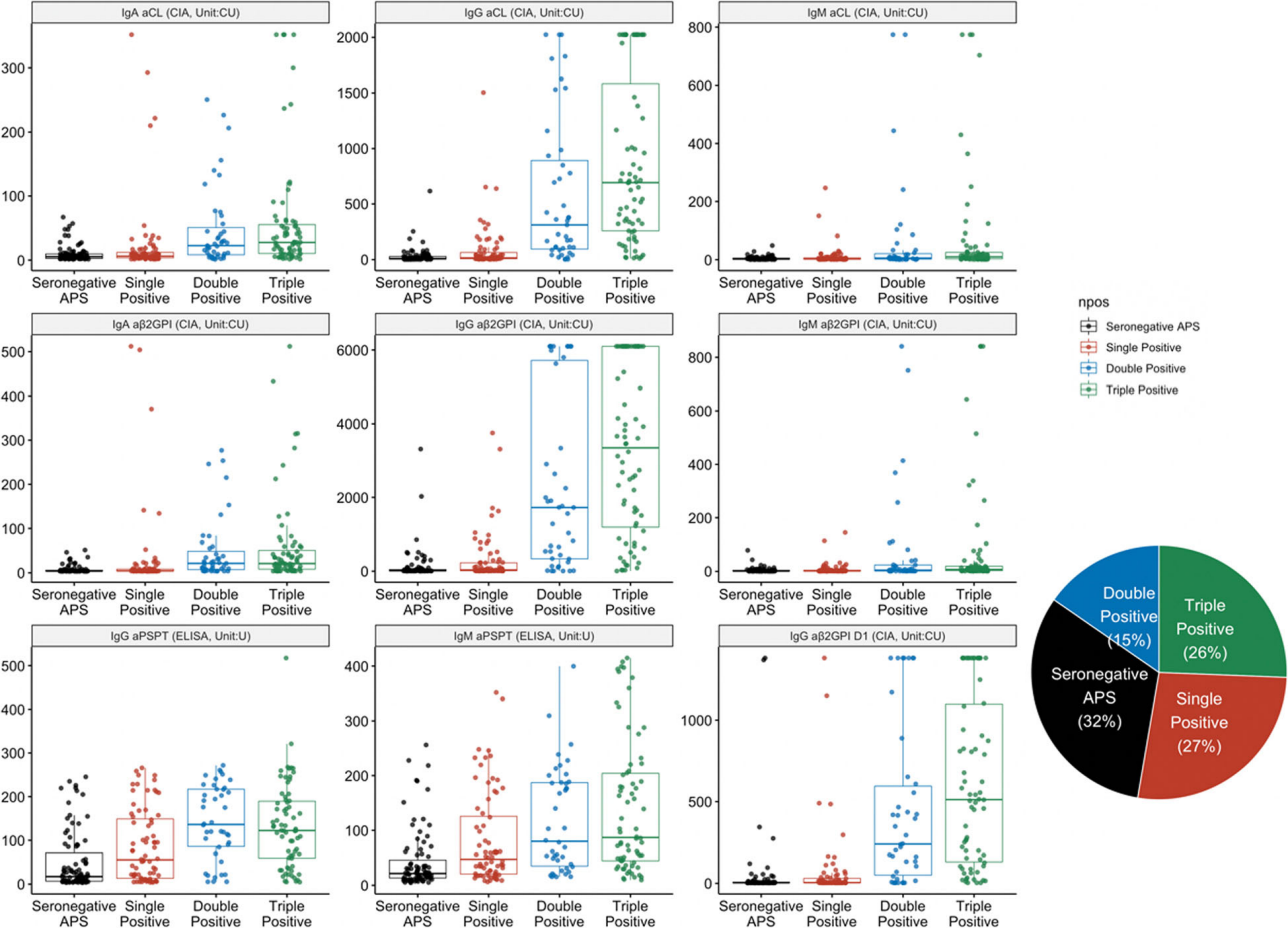
Serum titer of the aPLs grouped according to the number of positive criteria antibodies. Triple, double, and single refers to the positive numbers of the criteria aPLs. Positive aCL is defined as IgG aCL ≥ 40 GPL and/or IgM aCL ≥ 40 MPL by ELISA, positive aβ2GPI is defined as IgG aβ2GPI ≥ 20 SGU and/or IgM aβ2GPI ≥ 20 SMU, and positive LA is defined as ≥ 1.2, in line with the Sydney classification criteria

**Fig. 3:**
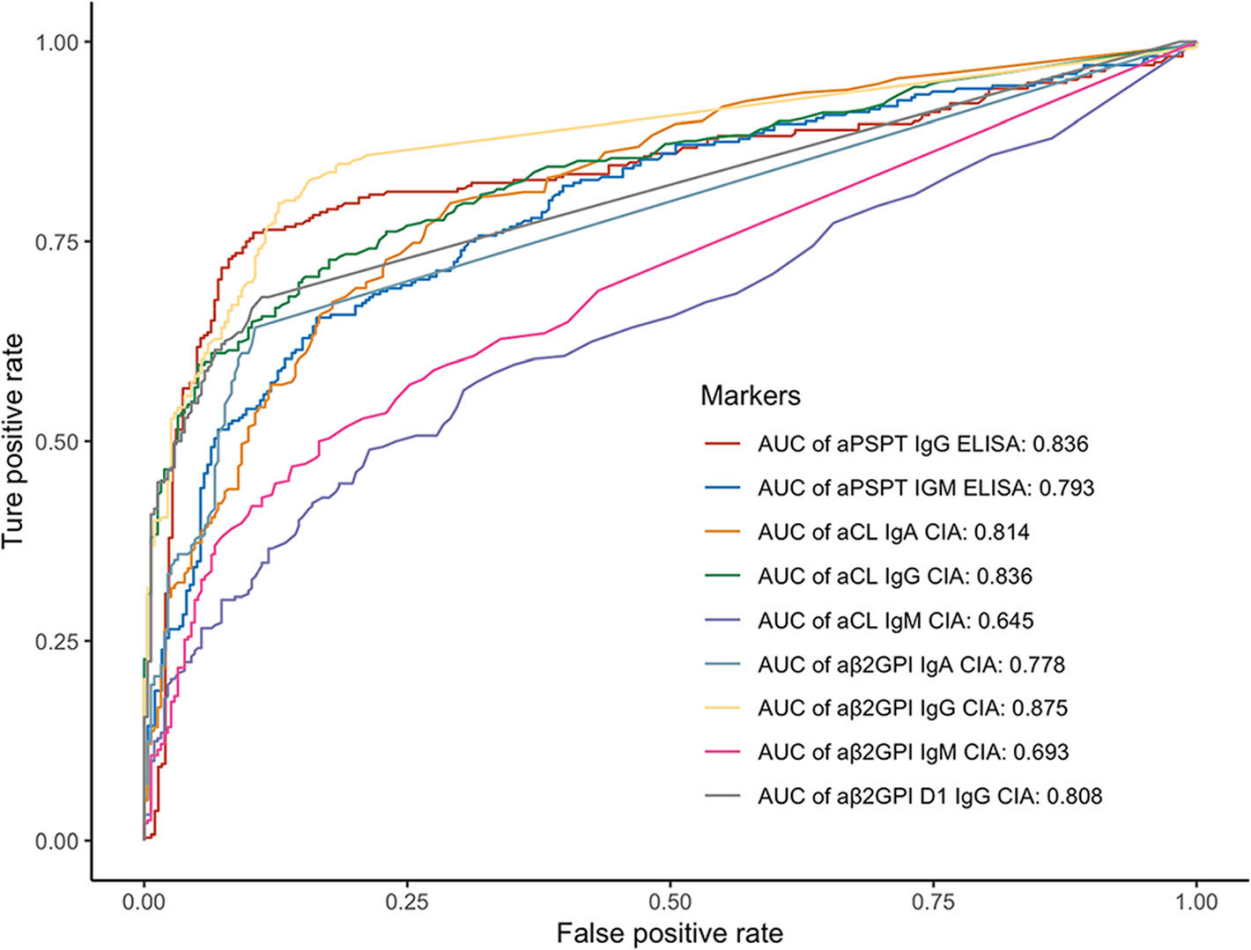
ROC plot of the individual aPLs

Distributions of the nine different criteria and non-criteria antibodies among the different clinical groups are portrayed in Fig. 1, and the frequencies of occurrence in is tabulated in Table 1. The serum levels of different aPLs are significantly elevated in the classic APS patients, while patients with SNAPS and SLE also have increased antibody levels compared with other disease controls or healthy controls. IgG isotypes of the antibodies show better reproducibility in the disease group and are more uniformly distributed compared with the IgA and IgM isotypes. The prevalence and titer of various aPL among the different subset of patients (SNAPS/single/double/triple positive) are illustrated in Fig. 2. Diagnostic values of the aPLs in APS and SNAPS patients were assessed by receiver operating characteristic curve (ROC) analysis (Fig. 3). Anti-β2GPI IgG showed the largest area under the curve (AUC, 0.875), followed by aPS/PT IgG (AUC, 0.836) and aCL IgG (AUC, 0.836).

### Prevalence of non-criteria aPLs in SNAPS group

**Fig. 4:**
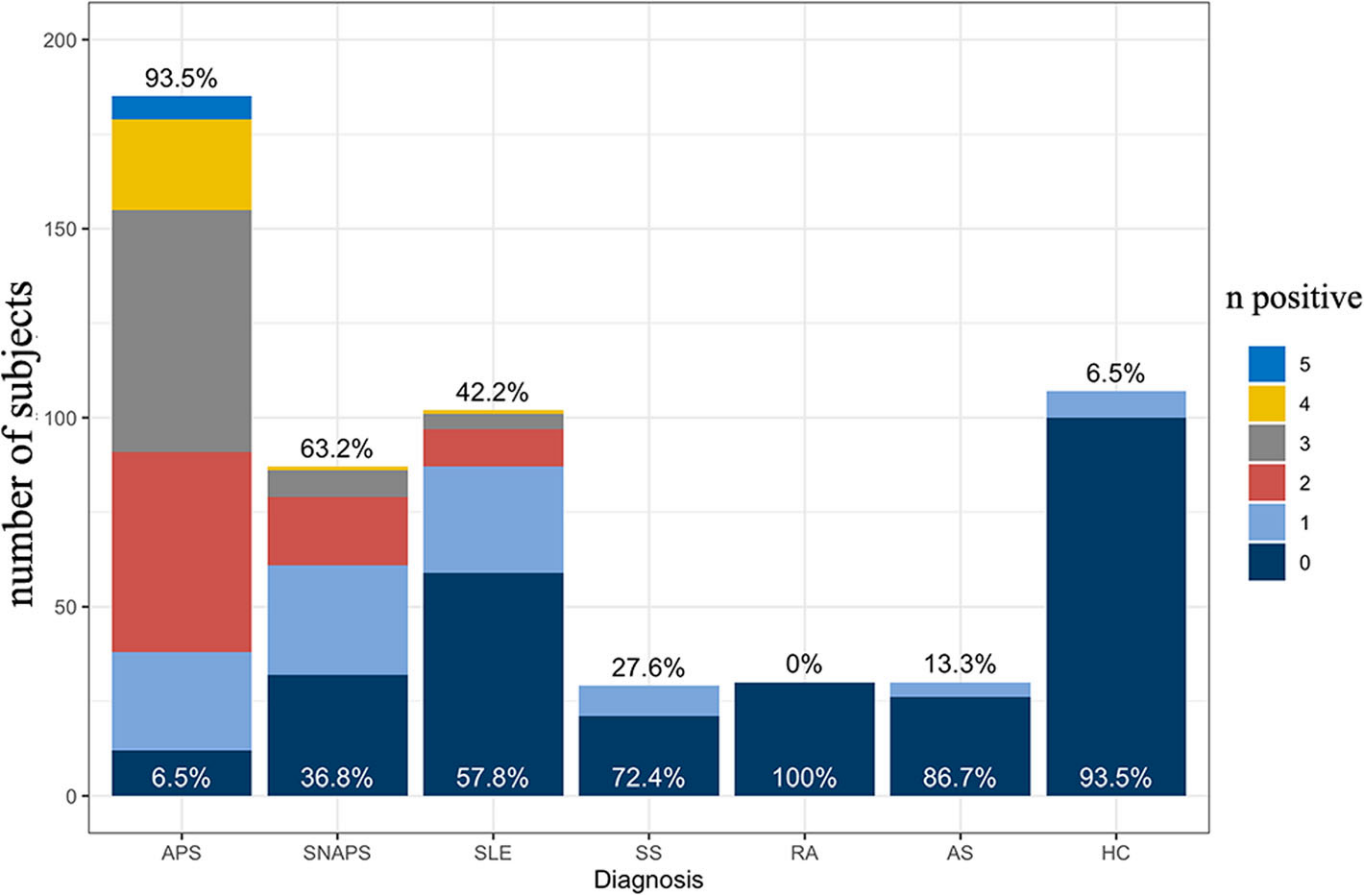
The number of the five non-criteria aPLs in different clinical groups. The black percentage above the bars indicates the percentage of patients with any positive non-criteria antibodies, while the white percentage on the bottom indicates those without positive non-criteria antibodies

The complementary ability of the non-criteria aPLs to identify SNAPS patients is among the most important aspects in the study of new antibodies. Figure 4 shows the number (from the set of aCL IgA, aβ2GPI IgA, aPS/PT IgG, aPS/PT IgM, and aβ2GPI D1 IgG) of positive non-criteria aPLs among the different clinical groups. 93.5% of the APS patients have at least one positive non-criteria antibody, a majority of these groups have three or more positive non-criteria antibodies, and 23.4% APS patients are positive for all five non-criteria aPLs. In the SNAPS and SLE groups, 60.9% and 40.8% of patients respectively are detected by non-criteria aPLs.

**Fig. 5:**
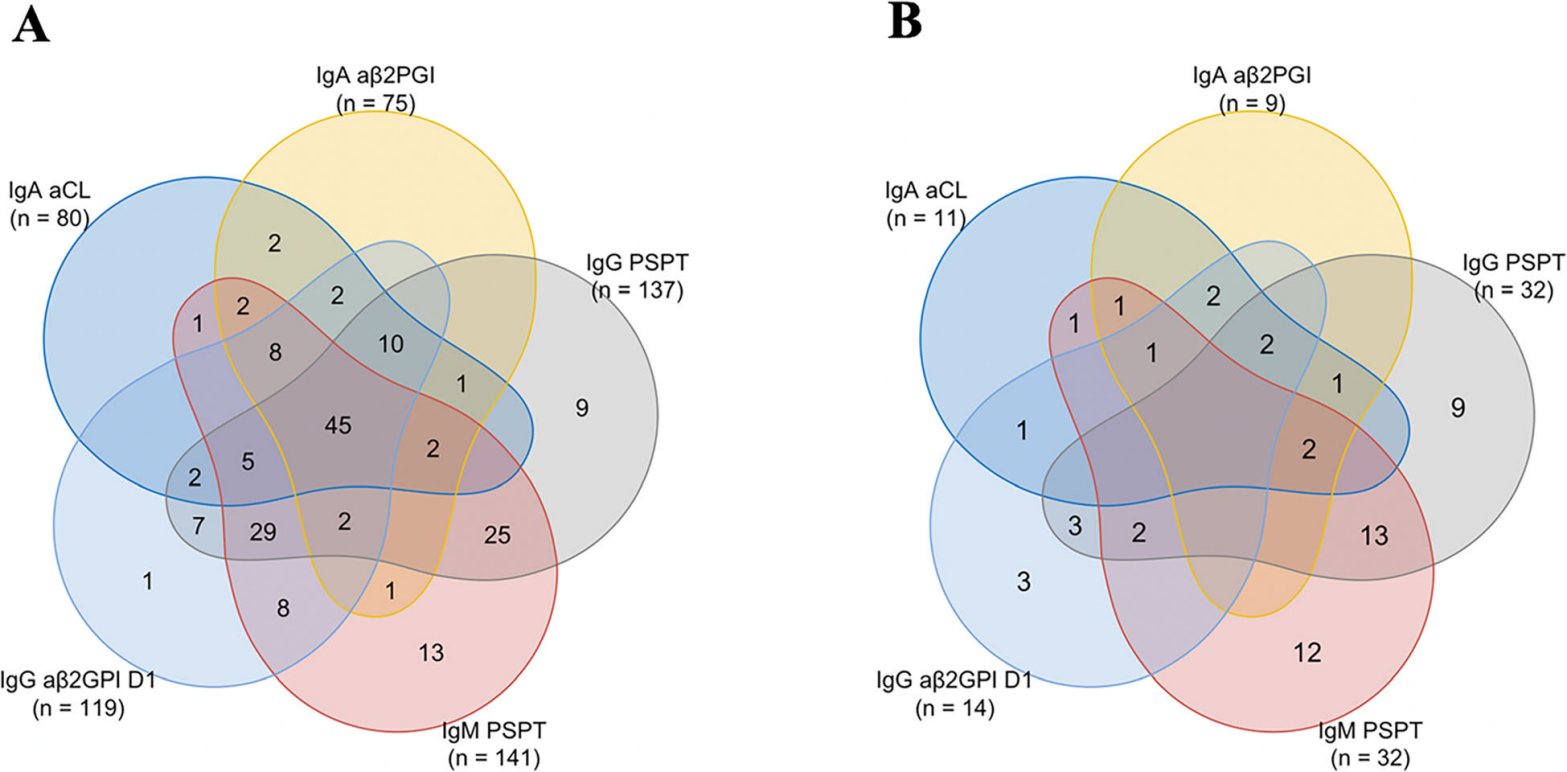
Venn plot revealing the multi-positive non-criteria aPLs in APS group (**a**) and in SNAPS group (**b**). Numbers in the overlapping region represent the number of patients with specific positive non-criteria aPLs patterns. Numbers in the non-overlapping region represent the number of patients with single positive non-criteria aPLs. Numbers in the brackets represent the number of patients in the group with positive outcome of the specific aPL

Additional analysis (Fig. 5) demonstrated that aPS/PT IgG and aPS/PT IgM are the most frequently detected aPLs in both APS and SNAPS patients. Also, the prevalence of aβ2GPI D1 IgG is higher in the SNAPS group than in the SLE group. It is important to note that aPS/PT IgG, aPS/PT IgM, and aβ2GPI D1 IgG can each be the only biomarkers detected in SNAPS patients, in contrast to aCL IgA and aβ2GPI IgA antibodies which tend to appear accompanied by other non-criteria aPLs.

### Relationship between clinical manifestations and criteria as well as non-criteria aPLs

**Fig. 6:**
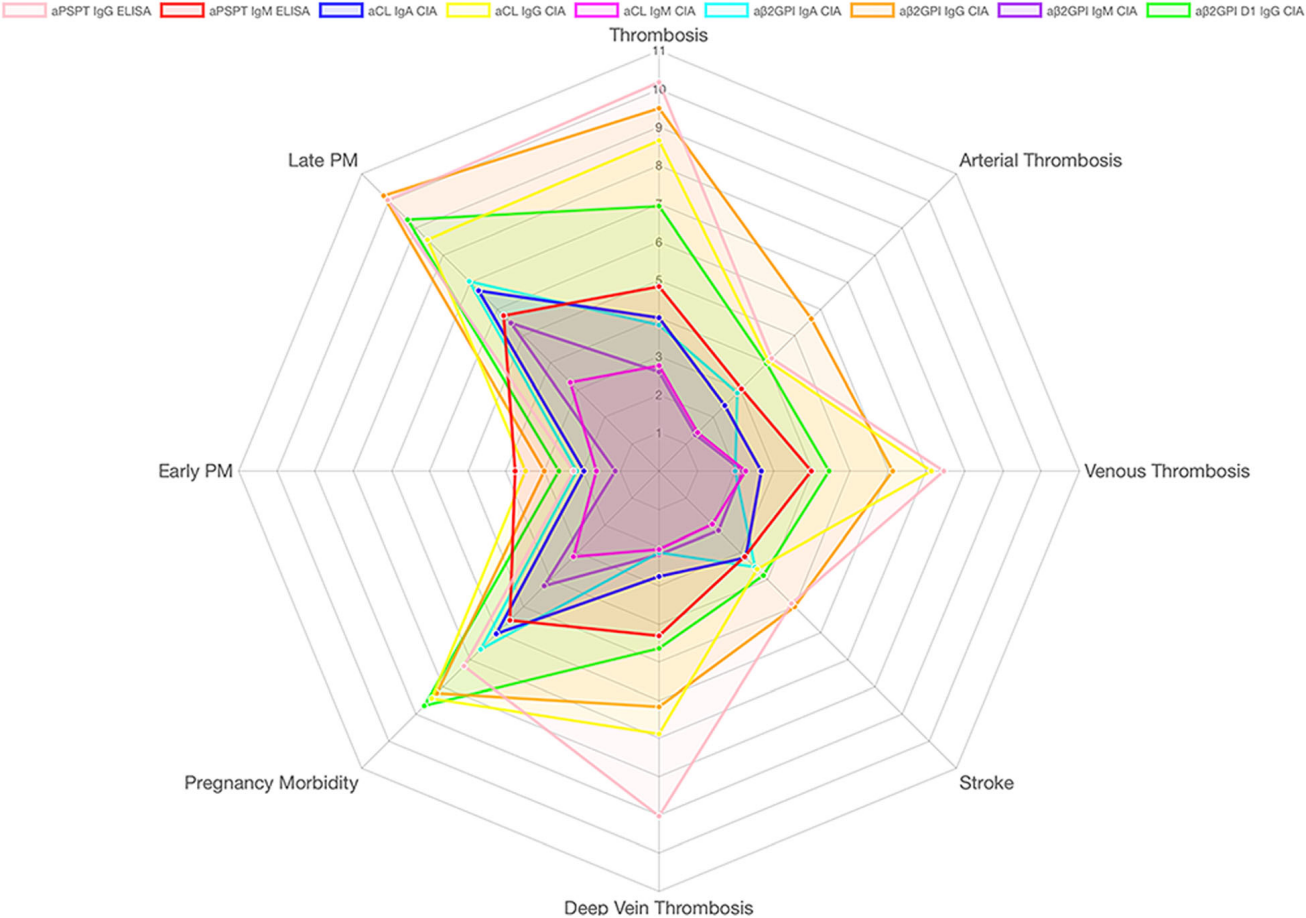
The predictive value of the aPLs in clinical affairs. Outcomes are presented in the form of odds ratios between positivity of the biomarker and the appearance of the APS-related clinical manifestations. The left part of the figure concerns pregnancy morbidities, in which “early pregnancy morbidity” refers to at least one fetal loss before 10 weeks of pregnancy, and “late pregnancy morbidity” refers to at least one stillbirth after 10 weeks of pregnancy or premature delivery before 34 weeks of pregnancy. All the outcomes have been adjusted with sex and age

To explore whether the non-criteria aPLs could add value for the prognostic prediction of clinical events, a radar plot (Fig. 6) depicting the odds ratios for clinical events was constructed. As can be seen from the figure, aβ2GPI IgG is the best predictor of arterial thrombosis with the odds ratio of 6.5 (95% CI, 3.64–8.75), while aPS/PT IgG with an odds ratio of 7.46 (95% CI, 4.69–11.88) is closely associated with venous thrombosis. Interestingly, stroke can be best predicted by aβ2GPI IgG, with the odds ratio of 5.01 (95% CI, 2.91–8.6), while aPS/PT IgG best predicts deep vein thrombosis with the odds ratio of 9.02 (95% CI, 5.5–14.81). With regards to early pregnancy morbidity, aPS/PT IgM shows the best association with an odds ratio of 3.78 (95% CI, 1.94–7.36). On the other hand, aβ2GPI IgG best predicts late pregnancy morbidity with an odds ratio of 10.21 (95% CI, 6.03–17.27). None of the odds ratios for the various non-criteria biomarkers are less than 1 in the analysis. Regardless of the detailed clinical manifestations, aPS/PT IgG and aβ2GPI Domain 1 IgG seem to be the most significant risk factors for thrombotic events and pregnancy morbidity, respectively.

From another perspective, IgG isotypes of aCL and aβ2GPI are better risk factors for the clinical manifestations of APS, followed by IgA aCL/aβ2GPI, while the IgM isotypes of the same aPL show the least association with the APS-related clinical events.

### Individual and combined values of the criteria and non-criteria aPLs in diagnosing APS and SNAPS patients

All combinations of the 10 biomarkers were enumerated and to find the best combinations of the aPLs, including LAC, to best define these groups of patients. Combinations of the biomarkers in all are analyzed, including 10 single markers. The performance of different aPLs was scored according to the Youden index and the OR. The results of the individual biomarkers and part of the combinations are shown in Table 2.

**Table 2:** Diagnostic values of the criteria and non-criteria aPLs

Parameters	Sen	Spe	PPV	NPV	PLR	NLR	OR (95% CI)	Youden index
LAC	0.4716	0.9808	0.9568	0.6732	24.6034	0.5387	45.67 (19.7–105.9)	1.4524
aCL IgA	0.3227	0.9617	0.8835	0.6118	8.417	0.7043	11.95 (6.37–22.41)	1.2844
aCL IgG	0.5993	0.9425	0.9037	0.723	10.421	0.4252	24.51 (14.4–41.73)	1.5418
aCL IgM	0.1525	0.9808	0.8776	0.5623	7.9545	0.8641	9.21 (3.85–21.99)	1.1333
aCL IgG/IgM	0.6277	0.9265	0.885	0.7342	8.5416	0.4019	21.25 (13.04–34.64)	1.5542
aCL IgA/G/M	0.6418	0.9105	0.866	0.7383	7.1749	0.3933	18.24 (11.54–28.84)	1.5523
aβ2GPI IgA	0.2979	0.9776	0.9231	0.6071	13.3191	0.7182	18.55 (8.4–40.92)	1.2755
aβ2GPI IgG	0.6702	0.9137	0.875	0.7546	7.7695	0.3609	21.53 (13.51–34.31)	1.5839
aβ2GPI IgM	0.1383	0.9776	0.8478	0.5574	6.1839	0.8814	7.02 (3.08–15.96)	1.1159
aβ2GPI D1	0.489	0.9712	0.9366	0.6862	17.0053	0.5262	32.32 (15.98–65.36)	1.4602
aβ2GPI IgG/M	0.6879	0.9073	0.87	0.7634	7.425	0.3439	21.59 (13.66–34.12)	1.5952
aΒ2GPI IgA/G/M	0.695	0.9073	0.8711	0.7676	7.5016	0.3361	22.32 (14.11–35.3)	1.6023
aΒ2GPI IgG/M/D1	0.6949	0.9042	0.863	0.7732	7.2496	0.3375	21.48 (13.61–33.9)	1.5991
aΒ2GPI IgA/G/M/D1	0.7022	0.9042	0.8643	0.7775	7.3263	0.3294	22.24 (14.08–35.14)	1.6064
aPSPT IgG	0.6213	0.9465	0.9135	0.7332	11.611	0.4001	29.02 (16.58–50.81)	1.5678
aPSPT IgM	0.636	0.8395	0.7828	0.7171	3.9619	0.4336	9.14 (6.15–13.57)	1.4755
aPSPT IgG/M	0.7978	0.8127	0.7949	0.8154	4.2597	0.2488	17.12 (11.31–25.92)	1.6105
aCL IgG/M/aΒ2GPI IgG/M/LAC	0.7872	0.8626	0.8377	0.8182	5.7303	0.2467	23.23 (15.11–35.71)	1.6498
aCL IgA/G/M/aΒ2GPI IgA/G/M/LAC	0.7943	0.8594	0.8358	0.8226	5.6505	0.2393	23.61 (15.36–36.3)	1.6537
aCL IgG/M/aΒ2GPI IgG/M/D1/LAC	0.7831	0.8594	0.8288	0.8201	5.5706	0.2524	22.07 (14.36–33.92)	1.6425
aPSPT IgG/M/aCL IgG/M/aΒ2GPI IgG/M/LAC	0.8897	0.7592	0.7707	0.8833	3.6948	0.1453	25.43 (16.01–40.4)	1.6489
aPSPT IgG/aCL IgA/aΒ2GPI IgA/G/LAC	0.8493	0.8763	0.8619	0.8647	6.863	0.172	39.9 (24.73–64.37)	1.7256
aPSPT IgG/aCL IgG/LAC	0.8235	0.8997	0.8819	0.8486	8.2078	0.1962	41.84 (25.65–68.26)	1.7232

As to the single biomarkers, aβ2GPI IgG had the best sensitivity of 0.6702, while the best specificity belongs to LAC with a value of 0.9808 in the current cohort. Overall, aPS/PT IgG appears to be a more balanced biomarker with the max Youden index of 1.5678 (sensitivity and specificity being 0.6312 and 0.9465, respectively). aβ2GPI Domain 1 IgG, which has been recognized as a pathogenic antibody of APS, has better specificity (0.9712) but much lower sensitivity (0.489) compared with the IgG antibody to the whole β2GPI molecule. aCL/aβ2GPI IgA possess nearly twice the sensitivity compared to the IgM isotypes of aCL/aβ2GPI, while maintaining similar specificity. Isolated lupus anticoagulant positivity has the highest OR of 45.67 (95% CI, 19.7–105.9), for the diagnosis of APS.

As to the combined analysis, the aPL combination with the largest Youden index is the combination of aPS/PT IgG, aCL IgA, aβ2GPI IgA, aβ2GPI IgG, and LAC, with a sensitivity and specificity of 84.93% and 87.63%, respectively, and an OR of 39.9 (95% CI, 24.73–64.37). The combination of aPS/PT IgG, aCL IgG, and LAC provides outstanding performance with a sensitivity of 82.35% and specificity of 89.97% with the least number of biomarkers.

In the next step, we rank the combinations according to the Youden indexes from largest to smallest and displayed the 10 aPL combinations with the biggest Youden indexes in Table 2. The frequency of occurrence of single biomarkers was added up for the sake of reproducibility. Anti-PS/PT IgG and LAC were present in all the combinations, supporting the robustness of the clinical value contributed by these two markers, followed by aβ2GPI IgG with an occurrence frequency of 0.9, and then IgA aβ2GPI and IgA aCL. Interestingly, aCL IgG is less often included in the best combinations, perhaps because of its strong correlation with other pathogenic antibodies.

## Discussion

While it has long been accepted that aPLs contribute to the pathogenesis of APS, the discordance between the persistent presence of aPLs (which may not fluctuate upon medical intervention) and isolated clinical adverse events has remained a continuous area of research focus. Discoveries of new aPLs have helped detect potential seronegative patients as well as provide additional insight into the mechanisms of the disease. Although there is a vast array of aPL biomarkers which have shown some indication of clinical or diagnostic value, most have not been replicated or well-validated outside of the research lab. In the present study, we chose five non-criteria aPLs, namely the IgA isotypes of aCL and aβ2GPI, aPS/PT IgG and IgM, and anti-β2GPI Domain 1 IgG (aβ2GPI D1 IgG) antibody. All five of these non-criteria aPLs can be detected in a considerable proportion of the SNAPS patients and could help physicians to identify patients with an elevated risk of thrombosis and pregnancy morbidity who may otherwise be missed.

Concerning the individual biomarkers, although the value of the IgA isotypes of the aPLs has been questioned because of varying prevalence among different study populations [37] and the heterogeneity between different assays [38], in the present study, the IgA isotypes of aCL/aβ2GPI far exceeded the IgM isotypes in sensitivity while maintaining very high specificity. Furthermore, the presence of anti-aPL IgA antibodies was related to a higher risk for thrombosis and pregnancy morbidity than the IgM aPLs. Although the prevalence of IgA aCL and IgA aβ2GPI was lower than the other non-criteria aPLs in both the APS and SNAPS group in our cohort, these markers can also provide additional hints for SNAPS diagnosis when other laboratory tests are unclear.

Several previous studies have suggested aPS/PT antibodies might be used as a surrogate for lupus anticoagulant detection [14] since the latter assay and its ancillary confirmatory assays is technically demanding and is not performed in many laboratories. Lupus anticoagulant remains a mysterious set of heterogeneous antibodies yet to be confirmed, while aPS/PT antibodies stand for an independent biomarker for APS and add value to SNAPS patients’ management.

Antibodies to β2GPI-D1, which are a subset of antibodies to the whole β2GPI molecule, showed a close relationship with pregnancy morbidities, especially with late pregnancy morbidity in our cohort, which is in accordance with a recently published retrospective study of obstetric patients [39].

In the combined analysis of the aPLs, adding the non-criteria aPLs separately or together added to the sensitivity of the total assay but simultaneously sacrificed the specificity, so maybe “the more the better” is not necessarily true in this case. Thus, in this study, the Youden index and odds ratio were used to evaluate the diagnostic performance of the antibody profiles. There is increasing recognition that scoring systems developed to quantify the contribution of different biomarkers and clinical manifestations (including the criteria and extra-criteria manifestations), as has been done in other rheumatic diseases. It is useful to evaluate and manage APS patients with the APS score and the Global APS Score in mind.

As to the relationship between aPLs and clinical manifestations, all of the biomarkers explored here are better predictors of late pregnancy morbidity rather than early pregnancy morbidity, supporting the concept that late pregnancy morbidity is a more specific clinical manifestation of the disease. Our results showed a higher prevalence of non-criteria aPL compared with similar studies previously reported [38, 40, 41]. On the one hand, variability between commercial diagnostic kits and lack of standardization among laboratories may account for the discrepancy. On the other hand, it perhaps reflects the specific makeup of our very well-characterized clinical cohort.

This study has some limitations. Histories of further clinical manifestations such as livedo reticularis, thrombocytopenia, valvular heart disease, epilepsy, and other manifestations related to aPLs may be included to better perform the analysis. As a result of the retrospective nature of our study, whether these aPL-positive patients will develop into APS is unknown and will be examined in a subsequent study.

APS can occur as an isolated diagnosis (primary APS), or it can be associated with systemic lupus erythematosus (SLE) and some of the criteria aPLs were included in the 1997 classification criteria for SLE of the American College of Rheumatology [32]. In clinical practice, APS is seen far more frequently in SLE or lupus-like disease than other autoimmune diseases. In the present study, a considerable proportion of the patients suffer APS secondary to SLE. On the other hand, some patients develop SLE several years after the manifestations of APS appear [42]; thus, while the relationship between APS and SLE is close, the factors leading to one condition or the other remain unclear. Close observation of patients with varying profiles of positive criteria, as well as non-criteria aPLs, may help understand the interval between serological positivity and the emergence of clinical manifestations.

## Conclusions

Recognition of SNAPS patients is critical for clinical management and prevention of potential thrombotic and obstetric adverse events. The non-criteria antiphospholipid antibodies help to identify a considerable portion (60.9%) of these patients who otherwise may remain untreated and at clinical risk. aPS/PT IgG and aβ2GPI Domain 1 IgG seem to be the most significant risk factors for thrombotic events and pregnancy morbidity, respectively.

## Data Availability

The datasets used and/or analyzed during the current study are available from the corresponding author on reasonable request.

## Abbreviations

APS: Antiphospholipid syndrome
aβ2GPI: Anti-β2-glycoprotein
aCL: Anti-cardiolipin antibodies
LAC: Lupus anticoagulant
SNAPS: Seronegative APS
aPS/PT: Anti-phosphatidylserine/prothrombin antibodies
aβ2GPI-D1: Anti-β2GPI Domain 1
aPLs: Antiphospholipid antibodies
CAPS: Catastrophic antiphospholipid syndrome
aPT: Anti-prothrombin antibodies
GAPSS: The Global APS Score
LBPA: Anti-lysobisphosphatidic acid
OR: Odds ratio
PAPS: Primary antiphospholipid syndrome
SAPS: Secondary antiphospholipid syndrome
SLE: Systemic lupus erythematosus
ACR: American College of Rheumatology
SLICC: Systemic Lupus International Collaborating Clinics
SS: Sjogren’s syndrome
AS: Ankylosing spondylitis
RA: Rheumatoid arthritis
HC: Healthy controls
PM: Pregnancy morbidities
CU: Chemiluminescent units
CI: Confidence interval
PPV: Positive predictive value
NPV: Negative predictive value
PLR: Positive likelihood ratio
NLR: Negative likelihood ratio
ROC: Receiver operating characteristic curve
AUC: Area under the curve
